# Development and validation to predict visual acuity and keratometry two years after corneal crosslinking with progressive keratoconus by machine learning

**DOI:** 10.3389/fmed.2023.1146529

**Published:** 2023-07-03

**Authors:** Yu Liu, Dan Shen, Hao-yu Wang, Meng-ying Qi, Qing-yan Zeng

**Affiliations:** ^1^Aier School of Ophthalmology, Central South University, Changsha, China; ^2^Aier Eye Hospital of Wuhan University, Wuhan, China; ^3^Aier Cornea Institute, Beijing, China; ^4^Aier School of Ophthalmology and Optometry, Hubei University of Science and Technology, Xianning, China

**Keywords:** crosslinking (CXL) corneal collagen, machine learning, keratoconus, prediction model, XGBoost (extreme gradient boosting)

## Abstract

**Purpose:**

To explore and validate the utility of machine learning (ML) methods using a limited sample size to predict changes in visual acuity and keratometry 2 years following corneal crosslinking (CXL) for progressive keratoconus.

**Methods:**

The study included all consecutive patients with progressive keratoconus who underwent CXL from July 2014 to December 2020, with a 2 year follow-up period before July 2022 to develop the model. Variables collected included patient demographics, visual acuity, spherical equivalence, and Pentacam parameters. Available case data were divided into training and testing data sets. Three ML models were evaluated based on their performance in predicting case corrected distance visual acuity (CDVA) and maximum keratometry (K_max_) changes compared to actual values, as indicated by average root mean squared error (RMSE) and R-squared (*R*^2^) values. Patients followed from July 2022 to December 2022 were included in the validation set.

**Results:**

A total of 277 eyes from 195 patients were included in training and testing sets and 43 eyes from 35 patients were included in the validation set. The baseline CDVA (26.7%) and the ratio of steep keratometry to flat keratometry (K_2_/K_1_; 13.8%) were closely associated with case CDVA changes. The baseline ratio of K_max_ to mean keratometry (K_max_/K_mean_; 20.9%) was closely associated with case K_max_ changes. Using these metrics, the best-performing ML model was XGBoost, which produced predicted values closest to the actual values for both CDVA and K_max_ changes in testing set (*R*^2^ = 0.9993 and 0.9888) and validation set (*R*^2^ = 0.8956 and 0.8382).

**Conclusion:**

Application of a ML approach using XGBoost, and incorporation of identifiable parameters, considerably improved variation prediction accuracy of both CDVA and K_max_ 2 years after CXL for treatment of progressive keratoconus.

## Introduction

Corneal collagen crosslinking (CXL) has been extensively used in clinical management of keratoconus since Wollensak et al. (1) originally demonstrated in 2003 that CXL enhances corneal stiffness. Although the effect of CXL in halting the progression of keratoconus has been widely recognized, long-term (≥ 2 years) randomized controlled (RCT) studies still indicated that its failure rate was highly variable (0–28%) (2–5) depending on CXL procedure type, age, race, disease severity, and other factors.

If CXL surgery fails, the progression of keratoconus continues, and CXL retreatment and/or keratoplasty are necessary. In China, there is a shortage of corneal donor tissue (6) which might induce patients to delay treatment. Meanwhile, this increases the financial and psychological burden on the patient. Therefore, accurate prediction of the postoperative outcome prior to CXL could help patients choose a newly alternative treatment options, such as intraocular lens or intrastromal implantation (7).

The most commonly used definition of keratoconus progression is maximum keratometry (K_max_) increase ≥ 1.0 D or corrected distance visual acuity (CDVA) decrease >2 lines (8). High preoperative K_max_, thin corneas thickness, and atopic diseases were widely recognized as the risk factors for progression of keratoconus after CXL (9–12). Female gender, young age, pronounced optical aberrations, and the cone location were also found as the risk factors of the progression in 2 years (10–12).

Several studies have utilized liner regression approaches to predict treatment outcomes including CDVA and K_max_, but these approaches failed to produce sufficiently reliable predictive power (13–17). An early study used a multivariate regression statistical model to predict CDVA and K_max_ for pediatric keratoconus patients (1 year follow-up) (13), and obtained a low predictive value model with CDVA (*R*^2^ = 0.45, *p* < 0.01) and K_max_ (*R*^2^ = 0.15, *p* > 0.05). A prior study (14) demonstrated that CDVA could potentially be predicted for 1 year postoperative following CXL, but did not evaluate the model’s ability to predict K_max_. Similarly, other studies only provided the predictors such as baseline K_max_ (15–17). However, the cornea biomechanics are not completely stable at 1 year following CXL.

Machine learning (ML) is a computer science discipline that utilizes algorithms and other approaches to automatically address complex problems that cannot easily be addressed by conventional data analysis means (18). Previous ML approaches have traditionally required very large datasets for training. With newly developed approaches, ML is now also suited for interrogation of small datasets with hundreds or dozens of variables using approaches such as few-shot learning (FSL) (19) and gradient boosting. The most widely used gradient boosting algorithms including categorical gradient boosting on decision trees (CatBoost), light gradient boosting machine (LightGBM), eXtreme gradient boosting (XGBoost), and Bayesian optimization (20).

Recently, ML has been used for keratoconus detection (21), classification (20), and candidacy for CXL treatment (23). However, no reports have used ML to predict the therapeutic outcome of CXL postoperatively. This study aimed to apply ML algorithms trained with a limited dataset to predict changes of visual acuity and keratometry 2 years following CXL for progressive keratoconus.

## Materials and methods

### Database

A retrospective medical chart review was conducted on all consecutive patients with progressive keratoconus who underwent CXL treatment between July 2014 and December 2020 at the Aier Eye Hospital of Wuhan University (Wuhan, Hubei province, China). Patients who returned for a follow-up visit at 2 years were included in the study. Data were collected using a convenient data management system supported by Empower Electronic Data Capture (EDC) system (https://empoweredc.com, Solution Inc., Shanghai, China).

An increase of at least 1 diopter (D) in maximum keratometry (K_max_) derived from computerized corneal topography during the preceding 12 months was required for inclusion. We enrolled keratoconus patients for all grades based on the Amsler-Krumeich keratoconus classification (24). Patients with previous refractive surgeries or corneal history of ocular surface or other eye disorders were excluded. In addition, patients whose data could not be reviewed for any reason were classified as being lost to follow-up and excluded from the study.

### Surgical technique

Patients were included regardless of their treatment protocols, which were not included in the prediction model. Two different treatment combinations were included in the study. When the thinnest corneal thickness of the eye was ≥ 450 μm, patients were undergo the high-fluence accelerated CXL (HF A-CXL). When the thinnest corneal thickness of the eye was < 450 μm, they were undergo the accelerated transepithelial CXL (A-TE CXL).

1. *A-TE CXL*: In the first step, 0.25% riboflavin (Paracel Part I, Avedro Inc., USA) containing 0.02% benzalkonium chloride (BAC) and 0.85% hydroxypropyl methyl cellulose (HPMC) was applied onto the cornea every 90 s for 4 min. Thereafter, part I solution was rinsed with 0.22% riboflavin (Paracel Part II, Avedro), and part II solution was instilled every 90 s over the next 6 min. UV-A was applied using the Avedro KXL System (Avedro Inc., Waltham, USA) with 30 mW/cm^2^ UV power for 8 min with a 1 s on/off cycle (7.2 J/cm^2^) (25).
2. *HF A-CXL*: The corneal epithelium was removed with a blunt knife in a 10 mm zone. CXL was then performed with 0.1% dextran-free riboflavin (VibeX Rapid, Avedro) instilled every 90 s for 10 min. Subsequently, it was placed under UA irradiation for 4 min at 30 mW/cm^2^ (7.2 J/cm^2^, Avedro) (26).

The operator verified irradiance prior to each treatment. The two CXL procedures are summarized in Table 1.

**Table 1:** Crosslinking treatment procedures.

Parameter	A-TE CXL	HF A-CXL
Fluence (total) (J/cm^2^)	7.2	7.2
Soak time and interval (minutes)	10 (1.5)	10 (1.5)
Intensity (mW/cm^2^)	30	30
Treatment time (minutes)	8	4
Irradiation mode (interval)	Pulsed (1 s on/1 s off)	Continuous
Epithelium status	On	Off
Riboflavin	ParaCel	Vibex Rapid

CXL = corneal crosslinking; A-TE CXL = accelerated transepithelial corneal crosslinking; HF A-CXL = high fluence corneal crosslinking.

### Pain medication and postoperative care

All patients received 0.5% levofloxacin drops four times daily for 3 days prior to surgery. Thirty minutes before surgery, patients received 2% pilocarpine (Sigma-Aldrich, St. Louis, MO, United States) and 0.4% oxybuprocaine hydrochloride (Bausch & Lomb Pty Ltd., NSW, Australia) drops three times, with 5 min between each administration.

At the end of the surgery, the corneal surface was dressed with a therapeutic soft contact lens (Bausch & Lomb Pty Ltd.) for at least 24 h until the epithelium had completely healed.

### Feature selection

Twenty-six preoperative variables were recorded in all patients: sex, age, uncorrected visual acuity (UCVA, logarithm of the minimum angle of resolution [LogMAR] units), CDVA (LogMAR units), spherical equivalence (SE), flat keratometry (K_1_), steep keratometry (K_2_), mean keratometry (K_mean_), astigmatism (Astig), eccentricity (ecc), maximum keratometry (K_max_), minimum corneal thickness (MCT), the most elevated points on the front corneal surfaces (F. Ele Th), the most elevated points on the back corneal surfaces (B. Ele Th), the index of surface variance (ISV), the index of vertical asymmetry (IVA), keratoconus index (KI), center keratoconus index (CKI), the index of height decentration (IHD), minimum radius of curvature (RMin), and Belin/Ambrósio final D value (BAD-D), which were measured by Pentacam (Oculus, Wetzlar, Germany). In addition, five incorporation parameters were also collected, including the ratio of K_2_ to K_1_ (K_2_/K_1_), the ratio of K_max_ to K_mean_ (K_max_/K_mean_), the ratio of K_max_ to K_1_ (K_max_/K_1_), the ratio of K_max_ to K_2_ (K_max_/K_2_), and the difference between K_max_ and K_mean_ (K_max-mean_). All the exams were executed by the skilled examiners. The ‘quality specifications (QS)’ was used to evaluate the quality of Pentacam images. If the QS is not ‘OK’, the exams were executed more than two times. Finally, at least two experienced ophthalmologists validated the accuracy of the images and data.

Features that demonstrated the highest feature importance to the model on primary runs were included for further analysis. Histograms of each numerical attribute were generated to understand the distribution features across distinct values. Categorical features (sex) were encoded into a binary representation to enable machine learning (ML) readability of algorithms.

### Model development

Patients who returned for a follow-up visit at 2 years before June 30, 2022 were included in the study to develop the model. Data processing and ML model development were performed in Python 3.9.7 using the *pandas* (version 1.3.4), *numpy* (version 1.20.3), and s*cikit-learn* (version 1.0.2; [mode: *sklearn.model_selection* and *sklearn.metrics*]) packages. Three models were run by supervised ML methods, while examples of inputs (features chosen) and outputs (actual changes in CDVA and K_max_) were provided to models as training inputs to build an algorithm for future predictions including, CatBoost, LightGBM, and XGBoost.

### Model evaluation and statistical methods

The database was randomly split into two groups: 80% of data (*n* = 222) was used for model training, while the remaining 20% of the dataset (*n* = 55) was reserved to test the model’s predicted case value for CDVA and K_max_ variation. The predicted values were compared to the actual case changes. Performance metrics included root mean squared error (RMSE) and *R*-squared (*R*^2^). Models were compared using a Nadeau and Bengio’s corrected resampled t-test (27, 28). Feature importance values were derived using prespecified methodology specific to the algorithms studied.

### Model validation

To validate the accuracy of the prediction model, we established a free website and used data from patients followed from July 2022 to December 2022. We use the same RMSE and *R*^2^ evaluations.

**Figure 1:**
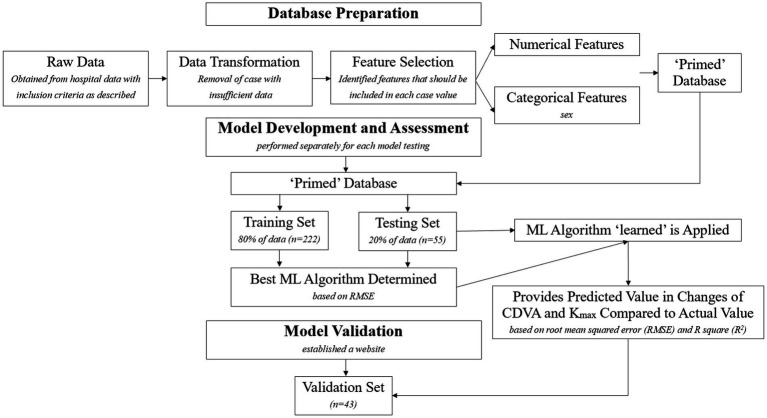
Workflow diagram detailing the data modeling process.

**Figure 2:**
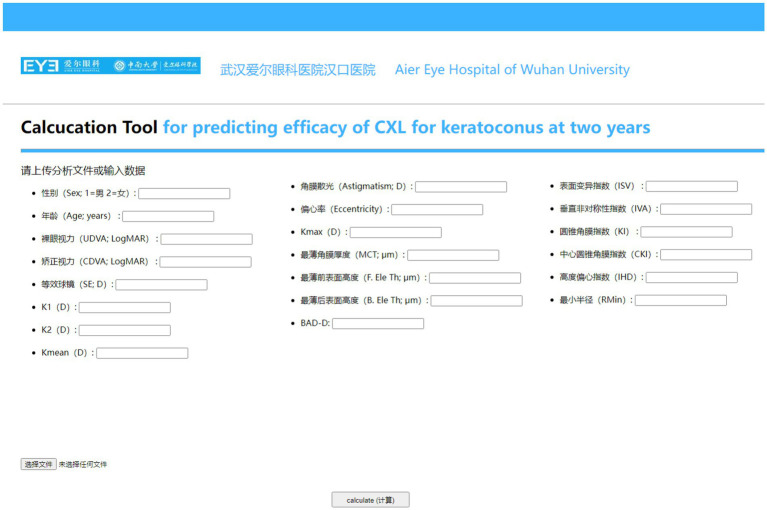
Online web-based calculator for predicting changes in CDVA and K_max_ 2 years after CXL crosslinking for keratoconus.

The workflow diagram detailing the data modeling process was showed in Figure 1. The website for validating the prediction model was showed in Figure 2.

## Results

### Patient characteristics

Data were recorded for 405 eyes from 289 patients who were diagnosed with progressive keratoconus and underwent CXL treatment. Thirty-nine patients (49 eyes) who were followed up at different clinics, and 30 patients (36 eyes) did not complete the 2 year follow-up, were excluded. Finally, the study included 277 eyes from 195 patients in training and testing sets and 43 eyes from 35 patients in the validation set. The demographic and baseline data of all patients are summarized in Table 2.

**Table 2:** Demographic and baseline data.

Parameter	Value in training and testing sets	Value in validation set
Patients	195 (151 males/44 females)	35 (28 males/7 females)
Eyes	277	43
Mean age (y, range)	20.09 (10 to 46)	20.31 (11 to 43)
UDVA (LogMAR)	0.71 ± 0.43	0.63 ± 0.44
CDVA (LogMAR)	0.27 ± 0.22	0.21 ± 0.19
SE (D)	−6.95 ± 0.22	−5.58 ± 3.04
K_1_ (D)	47.21 ± 4.59	45.17 ± 2.78
K_2_ (D)	51.12 ± 5.21	48.95 ± 3.96
K_mean_ (D)	49.05 ± 4.74	46.93 ± 3.11
Astig (D)	3.91 ± 2.36	3.78 ± 2.47
ecc	0.94 ± 0.27	0.83 ± 0.22
K_max_ (D, range)	58.13 ± 8.55 (44.1 to 83.4)	54.45 ± 5.82 (44.3 to 68.2)
MCT (μm)	454 ± 41	489 ± 29
F. Ele Th (μm)	23.77 ± 13.86	18.37 ± 9.73
B. Ele Th (μm)	52.71 ± 27.17	42.51 ± 20.41
BAD-D	9.62 ± 4.98	7.22 ± 3.1
ISV	88.64 ± 40.97	73.23 ± 31.54
IVA	0.83 ± 0.44	0.74 ± 0.42
KI	1.23 ± 0.14	1.18 ± 0.11
CKI	1.09 ± 0.08	1.06 ± 0.47
IHD	0.123 ± 0.075	0.098 ± 0.055
RMin	5.94 ± 0.82	6.27 ± 0.66
Follow-up (months, range)	24 (22 to 27)	24 (22 to 26)

UDVA = uncorrected distance visual acuity; CDVA = corrected distance visual acuity; LogMAR = logarithm of the minimum angle of resolution; SE = spherical equivalence; D = diopter; K_1_ = flat keratometry, K_2_ = steep keratometry, K_mea_ = mean keratometry; Astig = astigmatism; ecc = eccentricity; K_max_ = maximum keratometry; MCT = minimum corneal thickness; F. Ele Th = most elevated points on the front corneal surfaces; B. Ele Th = most elevated points on the back corneal surfaces; BAD-D = Belin/Ambrósio final D value; ISV = index of surface variance; IVA = index of vertical asymmetry; KI = keratoconus index; CKI = center keratoconus index; IHD = index of height decentration; RMin = minimum radius of curvature.

Data were presented as mean ± standard deviation or frequency.

### Clinical outcomes

Both CDVA and K_max_ improved significantly over baseline at the 2 year follow-up. Average CDVA decreased by 0.08 from 0.27 to 0.19 LogMAR (range: − 0.8 to 0.7 LogMAR; *p* < 0.001), and average K_max_ decreased by 1.06 D from 58.13 to 57.07 D (range: − 14.6 to 5.8 D; *p* < 0.001) in training and testing sets.

### Feature importance and model development

**Figure 3:**
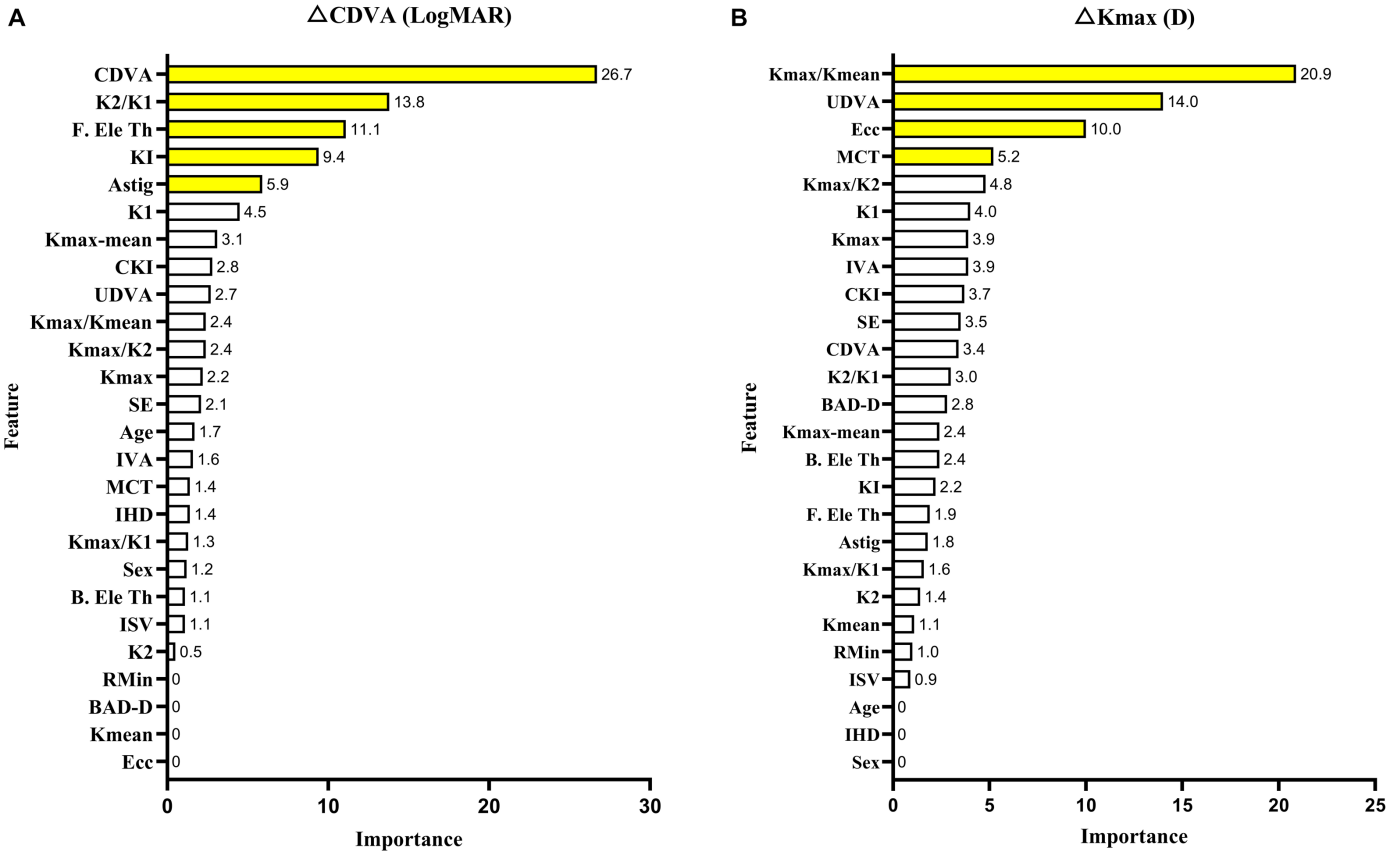
XGBoost model with the feature importance of each parameter in CDVA **(A)** and K_max_ **(B)** changes with keratoconus 2 years after crosslinking.

Feature importance was applied to select key features for improved performance. Baseline CDVA (26.7%), K_2_/K_1_ value (13.8%), and F. Ele Th (11.1%) were closely associated with case CDVA changes. The baseline K_max_/K_mean_ (20.9%), UDVA (14%), and ecc (10%) were closely associated with case changes of K_max_. The feature importance of each parameter is shown in Figure 3.

All baseline features were used as training features to construct a baseline regression model, to predict changes in CDVA and K_max_ 2 years after CXL while applying different algorithms. The XGBoost model demonstrated the best predictive ability in the training set (RMSE = 0.001 LogMAR and 0.013 D) compared to CatBoost and LightGBM. Therefore, XGBoost was selected for model and website building. Finally, our predictive model performed robustly in the testing set (*R*^2^ = 0.9991 and 0.9888). The performance of the three models in predicting changes of CDVA and K_max_ with the training and testing dataset were showed in Table 3.

**Table 3:** Performance of three models with changes of CDVA and K_max_ in training and testing data sets.

Dataset	Model	△CDVA (LogMAR)		△K_max_ (D)	
		RMSE	*R* ^2^	RMSE	*R* ^2^
Training Set	CatBoost	0.040	0.9410	0.089	0.9724
	LightGBM	0.031	0.9639	0.095	0.9682
	XGBoost	0.001	0.9998	0.007	0.9991
Testing Set	CatBoost	0.098	0.8892	0.504	0.8785
	LightGBM	0.066	0.9144	0.541	0.8719
	XGBoost	0.001	0.9993	0.053	0.9888

CatBoost = categorical gradient boosting on decision trees, LightGBM = light gradient boosting machine, XGBoost = eXtreme gradient boosting, △ = difference between 2-year post-CXL and pre-CXL; CDVA = corrected distance visual acuity; LogMAR = logarithm of the minimum angle of resolution; K_max_ = maximum keratometry; D = diopter; RMSE = root mean squared error; *R*^2^ = *R* square.

### Model validation

**Figure 4:**
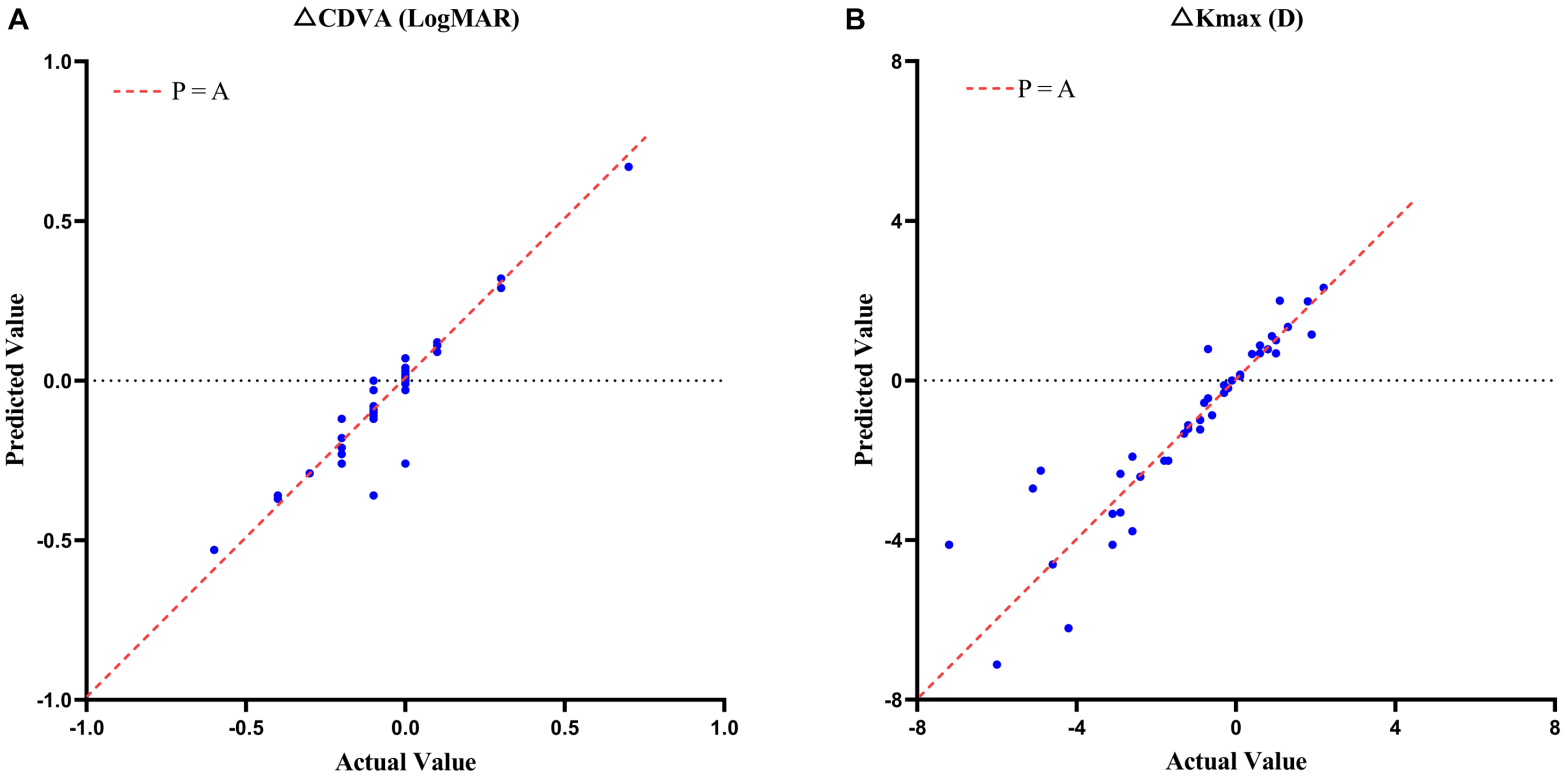
Scatterplots of the case predicted values compared to actual values in CDVA **(A)** and K_max_ **(B)** changes with keratoconus 2 years after crosslinking in validation set.

The CDVA also improved by 0.07 ± 0.21 LogMAR (*p* = 0.029) and K_max_ decreased by 1.16 ± 2.22 D (*p* = 0.001) in the validation set. The validation for the model achieved RMSE of 0.066 LogMAR and 0.907 D, and *R*^2^ of 0.8956 and 0.8382, respectively. The scatterplot of the predicted values compared to actual value in validation set were showed in Figure 4 and the raw data was showed in Supplementary Table 1.

### Adverse events

Opacity of the corneal stroma at the central and paracentral areas occurred in two eyes of one patients (10 years) during the follow-up in HF A-CXL treatment group. The minimum stromal thickness after epithelial removal were 411 and 429 μm. The patient had a history of sunlight exposure early in the postoperative period. Thereafter, the corneal transparency was restored after treatment with 0.1% fluorometholone (Allergan, Irvine, CA) and corneal protection to avoid direct irritation from sunlight (Supplementary Figure 1). No infections or other adverse events were observed in slit-lamp examination.

## Discussion

To our knowledge, this is the first study to use ML to predict the 2 year efficacy of CXL for keratoconus. Classical regression analysis has used one regression equation (linear regression) or several equations (hierarchical regression) to explain outcomes. The algorithm we ultimately used was XGBoost, which is accomplished through a process known as boosting. Boosting is an iterative procedure that intelligently adds weak learners to the ensemble model. The new weak learners will focus on the unlearned and thus strengthen the ensemble (20). By increasing the iterative over time, XGBoost improves the accuracy of regression analysis. Further, the XGBoost model will overfit when the dataset is too large, even if lasso or ridge regression are used to filter variables. Therefore, XGBoost is commonly used for small datasets and is effective for this application (29–31). The present study tested a variety of machine learning algorithms to develop prediction models for changes in CDVA and K_max_ 2 years following CXL for keratoconus, and XGBoost provided a superior models.

This research aimed to include five new incorporation variable indicators, including K_2_/K_1_, K_max_/K_mean_, K_max_/K_1_, K_max_/K_2_, and K_max-mean_. Further, some combinations of factors were relevant for improving model accuracy. Baseline CDVA was the most significant contributing feature in the CDVA change model, which is consistent with previous studies (13, 14, 32). The resultant feature importance could be simply defined as the extent to which the feature is incorporated into the model. In addition, we discovered that K_2_/K_1_ is another key feature in predicting CDVA changes. We hypothesized that K_2_/K_1_ was more important to CDVA changes than other indicators because CDVA monitors the overall visual function of the eye, which is directly connected to the shape of the cornea. Accordingly, the K_2_/K_1_ ratio is indicative of the general regularity of the front surface of the cornea. Some other combined parameters have been applied to develop a prediction model for keratoconus. A prior study indicated that the ratio of anterior radius of curvature (ARC) to posterior radius of curvature (PRC) was linked to CDVA 1 year after CXL for keratoconus (11). The greatest obstacle to correcting ametropia for keratoconus is irregularity of the cornea. A new parameter developed by Pabolo et al., the K-factor (K_F_ = K_2_ [K_2_–K_1_]) (33), was utilized to predict considerable improvement in CDVA after intracorneal ring segment implantation (ICRS). K_2_/K_1_ could determine the topographic form of the 3 mm of the central cornea, which contains the visual axis and is a crucial area for investigations focused on visual results. Similarity, KI and astigmatism (Astig) are two indicators of corneal regularity that can be used to evaluate corneal regularity, and account for 9.4 and 5.9%, respectively, of the feature importance outcomes in the CDVA change prediction model (Figure 3).

Concurrence of the highest elevated point on the front corneal surfaces (F. Ele Th) relative to the best fit sphere on the elevation maps was a novel important feature that could explain CDVA variations, consistent with a prior study (34). The study suggested that the difference in location between the most elevated areas on the corneal surfaces could be connected to biomechanical deterioration of the cornea. Corneal biomechanics could be connected to visual acuity, as the mechanical qualities of the cornea reflect its capacity to bear intraocular pressure. Furthermore, corneal curvature is intimately connected to visual acuity as described above.

According to prior studies, it is difficult to predict changes in K_max_ after CXL for keratoconus (13). This is most likely because K_max_, as measured by Pentacam, simulates corneal morphology and calculates maximum curvature *via* three-dimensional reconstruction of the collected corneal Scheimpflug pictures, rather than using directly measured values (35). Hence, K_max_ is influenced by an excessive number of variables. In the present study, we used a novel incorporation parameter, the baseline K_max_/K_mean_ ratio, to predict changes in K_max_. This parameter was much more predictive of final K_max_ than was baseline K_max_. This could be because baseline K_max_ is the maximum curvature of the anterior corneal surface, which indicates the absolute preoperative convexity of a point. However, after the crosslinking reaction, corneal rigidity increases, changing the biomechanical characteristics of the integrated cornea, thus there are additional factors impacting postoperative K_max_. Consistent with this notion, a recent study identified strong associations between corneal hysteresis (CH), corneal resistance factor (CRF), and K_max_ in keratoconic eyes, but not in crosslinked eyes (36). Variations in the biomechanics of the whole cornea result in more complex changes in K_max_ that are difficult to predict using preoperative data. Nevertheless, the K_max_/K_mean_ ratio maybe normalized for some unclear confounding factor, which reflects the convexity of the cornea, can be used to measure the overall qualities of the cornea.

It is presently unclear why baseline UDVA is a secondary-importance feature in the K_max_ change prediction model. One possible explanation is that visual function and corneal structure are inextricably linked (37).

Eccentricity (ecc) was identified as another significant predictive factor for changes in K_max_, consistent with prior findings (13, 15, 38). Additionally, central cones have a larger degree of postoperative corneal flattening than do peripheral cones (38). This conclusion could be explained by several factors. The efficacy of CXL is decreased in eccentric cones because UV devices cannot be applied uniformly across the treatment zone. UV rays could scatter in the perimeter, with a weaker and inconsistent beam in peripherally located cones. The second potential contributor is that even with homogenous light energy, the treatment power was relatively low in the peripheral cornea (39). Accordingly, cones in the periphery could be exposed to less crosslinking power, making ecc a significant predictor of K_max_ changes after CXL.

Minimum corneal thickness, the parameter of corneal thickness, was also of importance in predicting changes in K_max_ after CXL, which was consistent with an earlier study demonstrating a link between MCT and K_max_ variations (40). Corneal thinning in keratoconus results from defects in collagen lamellae caused by errors in the collagen lamellae manufacturing process (41). After stabilization with CXL, corneal collagen structural changes (42), primarily crimping (43), change corneal biomechanics. Therefore, using baseline data from MCT would enhance the prediction efficiency of K_max_.

Our pilot model still has several significant limitations that should be considered in its interpretation. First, even though XGBoost could effectively create a predictive model with a small dataset, our sample size was also limited, which could result in an unnecessarily complicated model. Second, parameters, such as atopic constitution, positive family history, and smoking, which did not have predictive potential in conventional linear regression analyses were excluded from the ML dataset (13, 16). However, XGBoost or other ML algorithms filter variables by lasso regression. The predictive potential of these parameters with ML approaches should be examined to determine their feature importance. Furthermore, a prior study found that keratoconus progression after CXL in one eye should be continuously monitored due to an increased chance of progression in the contralateral eye (44). This suggests that fellow eye data could also be incorporated into analysis.

Moreover, we found a pediatric patient who underwent HF A-CXL procedure has occurred the corneal opacity. Another study that used the same procedure to treat pediatric keratoconus patients did not report complications through 2 years postoperatively (45). Corneal haze following CXL has been reported in previous studies (46), but the reasons remain unclear at present. Potential reasons for this phenomenon are as follows: (1) more severe corneal ectasis caused by the fibroblast proliferation, which is more common in pediatric patients than in adults due to a more active proliferation response (47, 48); (2) the slow spontaneous crosslinking reactions triggered by residual riboflavin in the corneal stroma and UV-A rays in natural light (49); or (3) endothelial toxicity caused by reduced corneal thickness.

The study has several limitations that should be considered in its interpretation: the limited sample size, the multiplied CXL modalities, the various variable, the accuracy of pre-existing data, and the inherent biases introduced by retrospective analysis. Hence, these findings should be further confirmed by prospective trials with a longer follow-up period, larger sample size, and better variable selection.

Using an ML algorithm and incorporating identifiable parameters from historical case data improved prediction of case changes in CDVA and K_max_ at 2 years after CXL for progressive keratoconus. These techniques could improve case accuracy and decrease patient treatment expenses. To improve the prediction model, more data sets and richer feature collections should be examined in further studies.

## Data availability statement

The data analyzed in this study is subject to the following licenses/restrictions: The datasets used and analyzed during the current study are available from the corresponding author on reasonable request. Requests to access these datasets should be directed to Q-yZ, zengqingyan1972@163.com.

## Ethics statement

The studies involving human participants were reviewed and approved by Ethics Committee of Aier Eye Hospital of Wuhan University. Written informed consent to participate in this study was provided by the participants’ legal guardian/next of kin.

## Author contributions

YL conceived the study and was the major contributor in design and coordination, in collecting data, in analyzing data, in the web designing, and in writing the manuscript. DS and H-yW helped to collect the data. M-yQ made the web design. Q-yZ participated in surgeries and contributed in its design and coordination, and made the analysis and interpretation of data. All authors have read and approved the final manuscript.

## Funding

This study received support from the scientific research project of the Health Commission of Wuhan (Grant No. WX20Q19), and the Research Fund of the Clinical Research Institute of Aier Eye Hospital Group (No. AR2110D22).

## Conflict of interest

The authors declare that the research was conducted in the absence of any commercial or financial relationships that could be construed as a potential conflict of interest.

## Publisher’s note

All claims expressed in this article are solely those of the authors and do not necessarily represent those of their affiliated organizations, or those of the publisher, the editors and the reviewers. Any product that may be evaluated in this article, or claim that may be made by its manufacturer, is not guaranteed or endorsed by the publisher.
