# Supplementary material for: Development and validation to predict visual acuity and keratometry two years after corneal crosslinking with progressive keratoconus by machine learning
Source: Front Med (Lausanne). 2023 Jul 3;10:1146529. doi: 10.3389/fmed.2023.1146529 (PMC10393251; doi:10.3389/fmed.2023.1146529)
Supplement: Supplementary file 1 [file Table_1.DOCX]

**Supplementary Table - Case predicted and actual values by XGBoost model in changes of CDVA and K_max_ with keratoconus at 2-year after crosslinking in validation set**

|  | **△CDVA (LogMAR)** | |  | **△K_max_ (D)** | |
| --- | --- | --- | --- | --- | --- |
| **No.** | Predicted Value | Actual Value |  | Predicted Value | Actual Value |
| **1** | -0.09 | -0.1 |  | -2.71 | -5.1 |
| **2** | 0.29 | 0.3 |  | 1.15 | 1.9 |
| **3** | -0.12 | -0.1 |  | -4.12 | -3.1 |
| **4** | 0 | -0.1 |  | -2.26 | -4.9 |
| **5** | -0.099 | -0.1 |  | -6.21 | -4.2 |
| **6** | 0.67 | 0.7 |  | 2.32 | 2.2 |
| **7** | 0.01 | 0 |  | 0 | -0.1 |
| **8** | 0.03 | 0 |  | -4.61 | -4.6 |
| **9** | -0.11 | -0.1 |  | 0.11 | 0.1 |
| **10** | 0.09 | 0.1 |  | -1.33 | -1.3 |
| **11** | 0.11 | 0.1 |  | 0.68 | 1 |
| **12** | -0.18 | -0.2 |  | -1.91 | -2.6 |
| **13** | 0.02 | 0 |  | -3.31 | -2.9 |
| **14** | -0.21 | -0.2 |  | 0.66 | 0.4 |
| **15** | -0.29 | -0.3 |  | -2.41 | -2.4 |
| **16** | -0.08 | -0.1 |  | -0.99 | -0.9 |
| **17** | 0.04 | 0 |  | 0.15 | 0.1 |
| **18** | -0.21 | -0.2 |  | -7.12 | -6 |
| **19** | -0.09 | -0.1 |  | -0.45 | -0.7 |
| **20** | 0.01 | 0 |  | 0.88 | 0.6 |
| **21** | -0.01 | 0 |  | -1.21 | -1.2 |
| **22** | -0.29 | -0.3 |  | -3.34 | -3.1 |
| **23** | 0.32 | 0.3 |  | -3.78 | -2.6 |
| **24** | -0.23 | -0.2 |  | -2.01 | -1.8 |
| **25** | -0.12 | -0.1 |  | 1.11 | 0.9 |
| **26** | -0.1 | -0.1 |  | -2.34 | -2.9 |
| **27** | -0.03 | 0 |  | 0.68 | 0.6 |
| **28** | -0.26 | -0.2 |  | -0.56 | -0.8 |
| **29** | 0.02 | 0 |  | 1.34 | 1.3 |
| **30** | -0.08 | -0.1 |  | 1.01 | 1 |
| **31** | 0.07 | 0 |  | -1.12 | -1.2 |
| **32** | -0.03 | -0.1 |  | 0.67 | 0.6 |
| **33** | 0.07 | 0 |  | 2 | 1.1 |
| **34** | 0.12 | 0.1 |  | -0.19 | -0.2 |
| **35** | -0.1 | -0.1 |  | 0.79 | -0.7 |
| **36** | -0.37 | -0.4 |  | -1.23 | -0.9 |
| **37** | -0.1 | -0.1 |  | -0.12 | -0.3 |
| **38** | -0.53 | -0.6 |  | -0.87 | -0.6 |
| **39** | -0.12 | -0.2 |  | -2.01 | -1.7 |
| **40** | -0.36 | -0.1 |  | 1.98 | 1.8 |
| **41** | -0.36 | -0.4 |  | -0.31 | -0.3 |
| **42** | -0.26 | 0.0 |  | 0.78 | 0.8 |
| **43** | 0 | 0.0 |  | -4.12 | -7.2 |
| *△ = difference between 2-year post-CXL and pre-CXL; CDVA=corrected distance visual acuity; LogMAR = logarithm of the minimum angle of resolution; K_max_= maximum keratometry; D= diopter* | | | | | |
